# Spontaneous Coronary Artery Dissection in a Patient With a Single Coronary Artery

**DOI:** 10.7759/cureus.71647

**Published:** 2024-10-16

**Authors:** Ali Al-Shammari, Steven Danial Azmy Habib, Fredy Ramzy Shafik Gad

**Affiliations:** 1 Cardiology, Hampshire Hospitals Foundation Trust, Basingstoke, GBR; 2 Internal Medicine, Hampshire Hospitals Foundation Trust, Basingstoke, GBR; 3 Internal Medicine, University Hospitals of Leicester NHS Trust, Leicester, GBR

**Keywords:** : acute coronary syndrome, coronary artery anomaly (caa), non-st segment elevation myocardial infarction (nstemi), single coronary artery, spontaneous coronary artery dissection

## Abstract

Spontaneous coronary artery dissection (SCAD) is an uncommon non-atherosclerotic etiology of acute coronary syndrome (ACS) characterized by the formation of a false lumen inside the arterial wall, resulting in sudden occlusion of blood flow without any trauma or intervention. The pathogenesis of SCAD is not completely understood, and the association between coronary artery anomalies and SCAD remains unclear. This case study reports a unique occurrence of non-ST-elevation myocardial infarction (NSTEMI) in a 43-year-old female patient. NSTEMI is caused by spontaneous coronary artery dissection (SCAD) that affects the posterior descending artery (PDA) in the setting of a single coronary artery. This is a very rare subtype of coronary artery anomaly, in which the right coronary artery (RCA) gives rise to the left anterior descending artery (LAD) and left circumflex artery (LCx). The patient was managed conservatively, considering the extent of myocardial involvement and the resolution of symptoms. This congenital anomaly may have been the primary predisposing factor for SCAD development. Further research is required to determine the correlation between coronary artery anomalies and SCAD.

## Introduction

Spontaneous coronary artery dissection (SCAD) is a rare cause of acute, non-atherosclerotic coronary artery occlusion and accounts for between 0.2% and 4% of all acute coronary syndrome presentations [1]. It carries a similar risk of myocardial ischemia or infarction and related complications like fatal arrhythmias and heart failure [2]. SCAD can be idiopathic but more commonly associated with systemic diseases that increase the risk of vessel wall abnormalities, such as fibromuscular dysplasia and collagen and connective tissue diseases. Here, we present a case of SCAD in a woman with no systemic diseases who was found to have a rare subtype of coronary artery anomaly, which might be her main risk factor for developing spontaneous dissection.

## Case presentation

We report a case of a 43-year-old female patient who was admitted to our emergency department with typical chest pain. She developed an episode of acute central chest pain at rest, which radiated to her left arm with no associated nausea, vomiting, shortness of breath, palpitations, or diaphoresis. There were no preceding symptoms. She is normally fit and well, with no significant medical or surgical history. She has no family history of heart disease. She is a non-smoker and drinks alcohol occasionally. The initial assessment revealed a heart rate of 67 beats per minute, a blood pressure of 124/70 mmHg, a respiratory rate of 18 breaths per minute, and an oxygen saturation of 96% on room air. Her cardiovascular and respiratory examinations were normal. A 12-lead ECG revealed normal sinus rhythm with a normal PR interval, QRS complex, and ST segment. There were no dynamic ischemic changes in serial ECGs. Serial high-sensitivity troponin I levels showed a dynamic rise from 254 to 1383 ng/L within a span of six hours (normal for age <14 ng/L). A transthoracic echocardiogram revealed hypokinesia in the basal segment of the inferoseptum.

**Figure 1:**
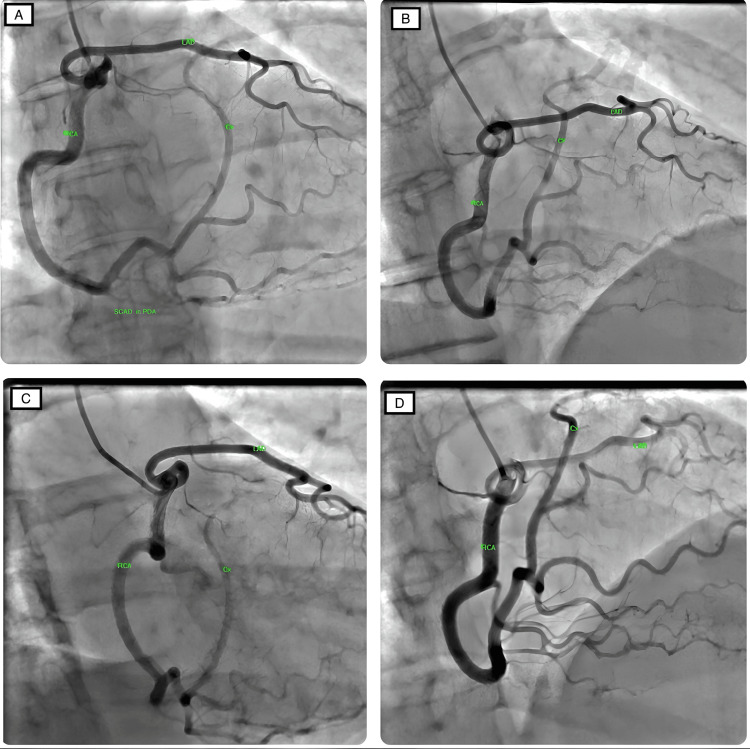
Coronary angiography demonstrating a single coronary artery originating from the right aortic sinus, and SCAD evident in PDA. LAD: left anterior descending artery; RCA: right coronary artery; Cx: circumflex artery; PDA: posterior descending artery; SCAD: spontaneous coronary artery dissection

In light of her clinical presentation and the dynamic rise in troponin I, she was diagnosed with non-ST-segment elevation myocardial infarction (NSTEMI). She was initiated on the acute coronary syndrome (ACS) treatment regimen in accordance with our trust guidelines. This included the administration of aspirin, ticagrelor, and fondaparinux, along with a plan to arrange a coronary angiogram and proceed with percutaneous coronary intervention if indicated. Coronary angiography was performed 24 hours later through the right radial approach using a 6-French radial sheath. We utilized the Tiger diagnostic catheter. We initially tried to engage the left coronary system but were unable to cannulate the left main stem (LMS) despite multiple attempts. The non-selective injection of contrast in the left coronary sinus did not reveal a coronary ostium in the left coronary sinus. Then, using the same Tiger catheter, we selectively cannulated the right coronary artery (RCA). This revealed that the entire coronary circulation originated from a single ostium within the right coronary sinus. The right coronary artery (RCA) gave rise to the left anterior descending (LAD) artery. The RCA then gave two branches: the posterior descending artery (PDA) and the posterolateral ventricular branch (PLV), which continued into the atrioventricular grove and became the anatomical circumflex artery. SCAD was noted in the PDA branch (Figure 1).

**Figure 2:**
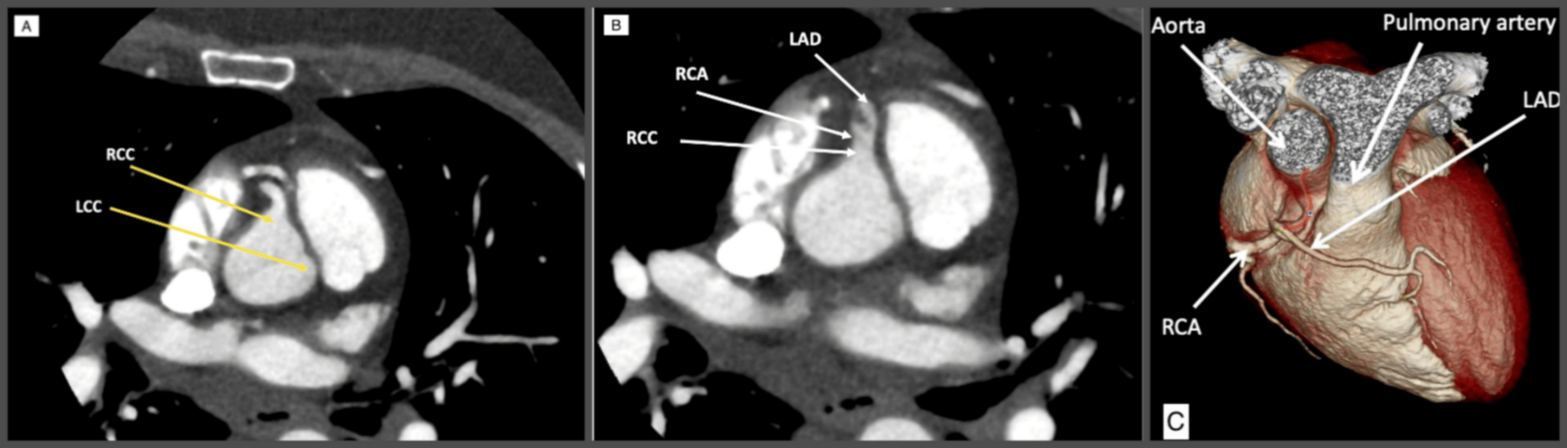
Computed tomography coronary angiography shows a single coronary artery originating from the right aortic sinus. LAD: left anterior descending artery; RCA: right coronary artery; LCC: left coronary cusp; RCC: right coronary cusp

After analyzing the angiographic findings and considering the affected artery and the extent of the myocardium implicated as seen in the echocardiogram, conservative management was decided with further investigations to delineate the coronary anatomy. A CT coronary angiography was done and revealed a solitary coronary artery arising from the right coronary sinus and giving rise to the left coronary artery, with no arteries emerging from the left coronary cusp (Figure 2).

After 48 hours of admission, the patient remained symptom-free and was discharged home with a plan for further follow-up in the cardiology clinic. The patient has been under follow-up in the cardiology clinic for two years, and there were no further acute presentations. She was also referred to the national SCAD center.

## Discussion

Spontaneous coronary artery dissection (SCAD) is a rare non-atherosclerotic cause of acute coronary syndromes (ACS). It is defined as an epicardial coronary artery dissection that is not iatrogenic and not associated with atherosclerosis or trauma [2]. Coronary artery obstruction occurs when an intramural hematoma forms, compressing the true lumen and obstructing the coronary flow distally. This is as opposed to the most common cause of coronary artery disease (ACS), which is atherosclerotic plaque rupture or intraluminal thrombus [2].

The coronary arteries consist of four layers: the luminal layer, tunica intima, tunica media, and tunica adventitia. Dissection occurs when blood accumulates in the tunica media, resulting in intramural hematoma formation. Blood originates from an injury to the vasa vasorum or an intimal tear. Regardless of how the intramural hematoma forms, it causes a blockage in the flow of blood to the coronary arteries because the hematoma expands. This results in coronary blood flow obstruction due to hematoma expansion, leading to myocardial infarction due to a supply-demand mismatch [3].

Historically, SCAD was thought to be a rare diagnosis, but recent series have reported that it is responsible for between 0.2% and 4% of all ACS cases. It is a significant factor contributing to ACS in young women, accounting for around 22.5% to 35% of all ACS cases in women under the age of 50 [1]. While the disease can impact people of all genders, it is notably more common in women, especially in their fifth and sixth decades of life, than in men [3].

The actual cause of spontaneous coronary artery dissection is not well established and is thought to be influenced by many factors. These patients had a lower prevalence of risk factors for atherosclerotic coronary artery disease compared to those who suffer acute coronary syndrome (ACS) due to plaque rupture. The established risk factors for SCAD include pregnancy, postpartum state, arteriopathies such as fibromuscular dysplasia (FMD), chronic inflammatory conditions like systemic lupus erythematosus (SLE), rheumatoid arthritis (RA), inflammatory bowel disease (IBD), sarcoidosis, connective tissue disorders like Marfan syndrome and Ehlers-Danlos syndrome, as well as physical and emotional stressors [1]. 

Invasive coronary angiography is the most preferred modality for diagnosing SCAD, irrespective of its first presentation. Intravascular ultrasonography is an alternative method used to diagnose SCAD, particularly when invasive angiography is inconclusive or contraindicated. Computed tomography coronary angiography (CTCA) is a non-invasive imaging technique that provides clear visualization of the artery wall and lumen. However, it is not considered the first-line diagnostic approach for SCAD [3]. 

Interestingly, coronary artery anomalies are not known causes of SCAD, and their association remains unclear owing to scarce available data. Yang et al. reported a patient with single sinus origin of all coronary arteries who developed SCAD, and they brought attention to this concept. However, it is important to note that other risk factors were also identified in this particular patient [4]. Rabbani et al. also presented a patient with SCAD in a single anomalous RCA arising from the left coronary sinus, highlighting the potential association between SCAD and coronary artery anomalies in the absence of any other known risk factors [5].

Single coronary artery (SCA) is a very uncommon congenital abnormality, occurring in around 0.024% to 0.066% of patients who undergo routine coronary angiography [6,7]. It can be associated with other congenital heart diseases but also can be detected as an isolated anomaly in a structurally normal heart [6]. 

Single coronary artery anomalies were classified based on the site of origin, anatomical course, and relationship of the coronary artery to the aorta and pulmonary artery using the Lipton et al. classification system [8]. Yamanaka and Hobbs later modified this system to include an additional feature, its septal course [9].

The single coronary artery is initially identified as "R" or "L" based on the location of the ostium in either the right or left sinus of Valsalva. Accordingly, it is classified as group I, II, or III. Group I follows an anatomical path along either the right or left coronary artery. Group II originates from the proximal segment of the normally located right or left coronary artery. Group III characterizes the abnormality in which the LAD and circumflex artery (CX) originate independently from the proximal segment of the normally located right coronary artery. The last classification pertains to the correlation between the abnormal coronary artery and the aorta and pulmonary artery. The letters "A," "B," "P," "S," and "C" denote the anterior, between, posterior, septal, and combined patterns, respectively, to provide a more exact and accurate description of anatomical deviations [8,9]. 

Conventional coronary angiography is the most reliable method for identifying the coronary anatomy. Recently, computed tomography coronary angiography (CTCA) has gained popularity due to its ability to accurately display the intricate structure of the coronary arteries and its non-invasive nature. 

**Figure 3:**
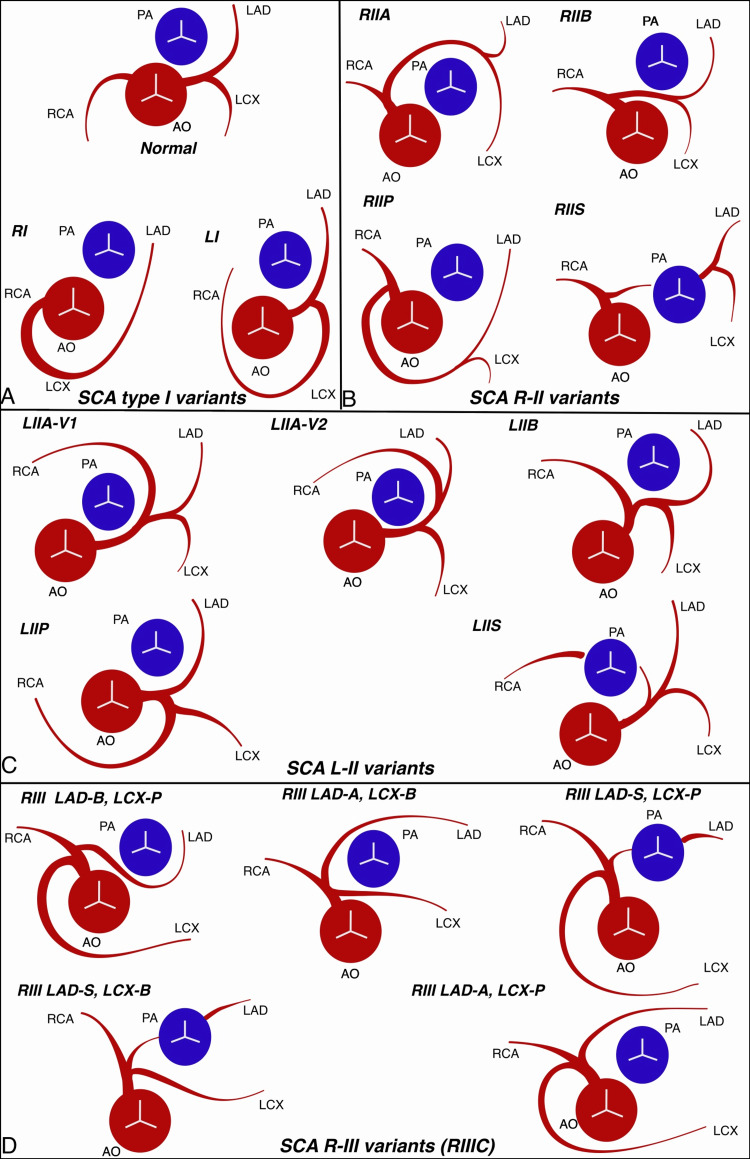
Single coronary artery classification. This diagram is an original work of the authors. Lipton-Yamanaka classification of single coronary artery, including variants proposed by Sampath et al. [10,11]. PA: pulmonary artery; AO: aorta; RCA: right coronary artery; LAD: left anterior descending artery; LCX: left circumflex artery; LMS: left main stem A: The illustration depicts the normal coronary anatomy, in which the right and left coronary arteries originate from the respective coronary sinuses. Additionally, it depicts a single coronary artery, specifically the right type I (SCA RI) and left type I (SCA LI), which originate from the respective coronary sinuses and supply the entire myocardium. B: Illustrates the different variations of the single coronary artery, right type II (SCA RII): RIIA (where the LMS travels anterior to the pulmonary artery), RIIB (where the LMS travels between the aorta and pulmonary artery), RIIP (where the LMS travels posterior to the aorta), and RIIS (where the LMS goes through the interventricular septum). C: Illustrates the different variations of the single coronary artery, left type II (SCA L-II): LIIA (LIIA-V1 and LIIA-V2; RCA originating from the LMS or LAD and traversing anterior to the pulmonary artery), LIIB (RCA passing between the aorta and pulmonary artery), LIIP (RCA passing posterior to the aorta), and LIIS (RCA passing through the interventricular septum). D: Illustrates the different variations of single coronary artery, right type III (SCA R-IIIC), where the RCA divides into LAD and LCX with no LMS. These branches follow combined courses according to the classification outlined by Yamanaka et al. [9].

Based on the coronary angiogram and CT coronary angiogram findings, our case has been classified as Lipton’s R-IIIC subtype anomaly (Figure 3). However, it did not precisely match any of the RIII variations described by Sampath et al. [10].

The primary pathological process that led to this patient's presentation was a spontaneous coronary artery dissection (SCAD) of the posterior descending artery (PDA) branch of the right coronary artery (RCA). Although the dissection only affected a minor branch, it was sufficient to elicit ischemia symptoms and some degree of left ventricular regional wall motion abnormalities. As the patient does not have any significant past medical history, either risk factors for SCAD or atherosclerotic coronary artery disease, this has raised the possibility that having the coronary anomaly might have contributed to the development of SCAD. 

## Conclusions

This case presents a rare subtype of coronary artery anomalies, as well as the development of SCAD, which is a rare cause of ACS. The etiology of SCAD is still unclear, and its association with coronary artery anomalies is yet to be ascertained. Additional studies are required to investigate this association further.
